# Projective Measurement-Based Quantum Phase Difference Estimation Algorithm for the Direct Computation of Eigenenergy Differences on a Quantum Computer

**DOI:** 10.1021/acs.jctc.3c00784

**Published:** 2023-10-24

**Authors:** Kenji Sugisaki

**Affiliations:** † Graduate School of Science and Technology, Keio University, 7-1 Shinkawasaki, Saiwai-ku, Kawasaki, Kanagawa 212-0032, Japan; ‡ Quantum Computing Center, Keio University, 3-14-1 Hiyoshi, Kohoku-ku Yokohama, Kanagawa 223-8522, Japan; § Centre for Quantum Engineering, Research and Education TCG Centres for Research and Education in Science and Technology, Sector V, Salt Lake, Kolkata 700091, India

## Abstract

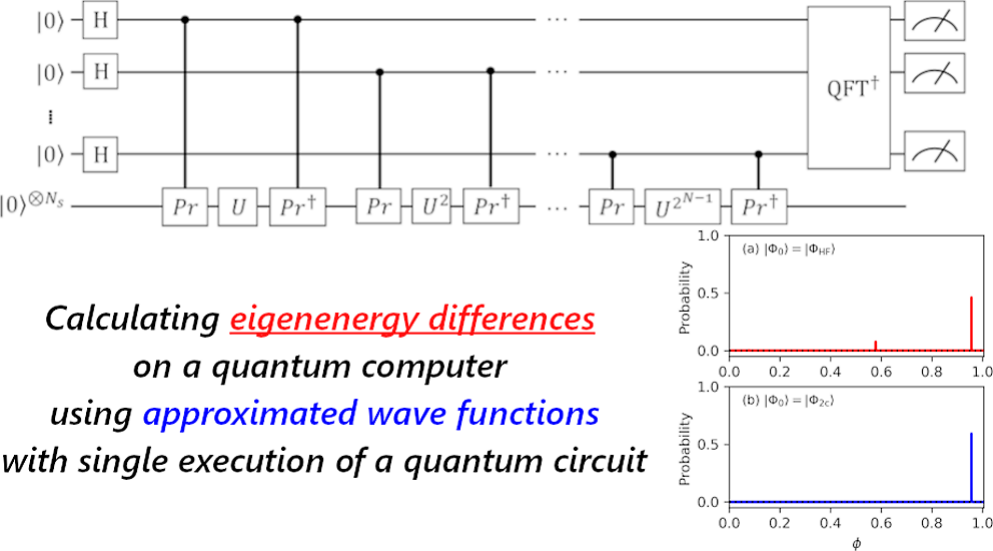

Quantum computers are capable of calculating the energy difference of two electronic states using the quantum phase difference estimation (QPDE) algorithm. The Bayesian inference-based implementations for the QPDE have been reported so far, but in this approach, the quality of the calculated energy difference depends on the input wave functions being used. Here, we report the inverse quantum Fourier transformation-based QPDE with *N*_a_ of ancillary qubits, which allows us to compute the difference of eigenenergies based on the single-shot projective measurement. As proof-of-concept demonstrations, we report numerical experiments for the singlet–triplet energy difference of the hydrogen molecule and the vertical excitation energies of halogen-substituted methylenes (CHF, CHCl, CF_2_, CFCl, and CCl_2_) and formaldehyde (HCHO).

## 1 Introduction

Quantum computing is one of the most innovative research fields in current science and is anticipated to bring breakthroughs in quantum chemical calculations. Quantum chemical calculations are based on the Schrödinger equation, which governs the dynamics of quantum particles, and accurate quantum chemical calculations can potentially open the door to predictive quantum chemistry. However, as Dirac noted, “The underlying physical laws necessary for the mathematical theory of a large part of physics and the whole of chemistry are thus completely known, and the difficulty is only that the exact application of these laws leads to equations much too complicated to be soluble”.^1^ The computational cost of the full configuration interaction (full-CI) method, which is the variationally best possible wave function in the Hilbert space spanned by the basis set used, scales exponentially with system size, and it is impractical except for small molecules with a medium-size basis set.

Since quantum chemical calculations deal with the dynamics of electrons in atoms and molecules, it is a potentially amenable problem for quantum computers. In fact, in 2005, Aspuru-Guzik and coworkers reported a method for performing the full-CI calculation on a quantum computer,^2^ using a quantum phase estimation (QPE) algorithm.^3–5^ The QPE is a quantum algorithm that is capable of computing the eigenvalues and corresponding eigenvectors of a unitary operator *U*. By using the time evolution operator exp(−*iHt*) for *U*, one can calculate the full-CI energies on a quantum computer. However, because QPE utilizes the projective measurement of the quantum state to obtain the eigenfunction and the corresponding eigenvalue, it is probabilistic, and which electronic state is obtained from the QPE depends on the overlap between the approximate wave function used as the input and the exact eigenfunction. Importantly, the QPE itself does not guarantee an exponential speedup of quantum chemical calculations,^6^ and connecting theoretical methods to generate sophisticated approximated wave function is necessary.

Quantum chemical calculations can afford to compute the total energies of atoms and molecules, but total energies are generally not available from experiments. Chemical phenomena relevant to quantum chemical calculations are usually discussed in terms of the energy differences between different geometries or electronic states. Thus, an accurate prediction of the energy differences is crucial for the practical use of quantum chemical calculations in chemistry research and development.

Because quantum computers can use quantum superposition states as computational resources, it is possible to compute the energy differences directly on a quantum computer. In fact, several quantum algorithms for the direct estimation of energy differences have been reported, such as the quantum annealing-based approach,^7^ the quantum–classical hybrid algorithm,^8^ using the robust phase estimation technique,^9^ and the algorithms for fault-tolerant quantum computers including the Bayesian exchange coupling parameter calculator with broken-symmetry wave functions (BxB) algorithm^10^ and the Bayesian phase difference estimation (BPDE) algorithm.^11–14^ In addition to these methods, multiple eigenvalue estimation techniques for the simultaneous determination of the ground and the excited-state energies have been reported.^15–17^ In particular, the BPDE algorithm reported by us is an extension of the Bayesian phase estimation (BPE)^18–20^ to the quantum phase difference estimation using the quantum superposition of two electronic states. BPDE is in principle applicable to arbitrary electronic states, and it is free from the controlled-time evolution requited in conventional QPE algorithms for total energy calculations. The BPDE algorithm has been applied to the direct calculation of ionization energies, singlet–triplet energy differences and valence excitation energies,^11^ total energies by using the quantum superposition of the desired electronic state and the vacuum state,^12^ finite difference-based numerical energy gradients,^13^ and relativistic energy differences (fine structure splitting).^14^ The BPDE algorithm is a powerful tool for studying the energy difference of atoms and molecules, but it has two major weaknesses. (1) It requires many circuit executions to compute the energy differences, and the calculated energy differences suffer from shot noise; and (2) it is not projective, and the calculated energy difference depends on the approximated wave functions used as the input. The second drawback can be clearly seen in the total energy calculations of the transition state between *trans* and *iso* isomers of the N_2_H_2_ molecule.^12^ It is desirable to extend the quantum phase difference estimation (QPDE) algorithm to projective measurement-based methods.

In this work, we propose a projective measurement-based QPDE algorithm with the *N*_a_ of ancillary qubits. Hereafter, we denote the proposed approach as the “*N*-qubit QPDE” algorithm. The *N*-qubit QPDE is a natural extension of the *N*-qubit QPE to the phase difference estimation, and it can compute the eigenenergy difference even if we use approximated wave functions as inputs. Similar to the *N*-qubit QPE for total energy calculations, which eigenenergy difference is obtained in the *N*-qubit QPDE depends on the overlap between the approximated wave functions and the eigenfunctions. To our knowledge, all other approaches for the direct calculation of the energy difference reported so far are neither projective nor single-shot protocols, and this is the first attempt to perform the eigenenergy difference calculation on a quantum computer without repetitive quantum circuit executions. As the demonstrations, we report numerical quantum circuit simulations for the singlet–triplet energy difference of the H_2_ molecule and the vertical excitation energies of halogen-substituted methylenes (CHF, CHCl, CF_2_, CFCl, and CCl_2_) and formaldehyde (HCHO). We also report the application of an algorithmic error mitigation (AEM)^21^ to remove the Trotter–Suzuki decomposition error and to improve the calculated energy differences.

## 2 Theory

**Figure 1:**
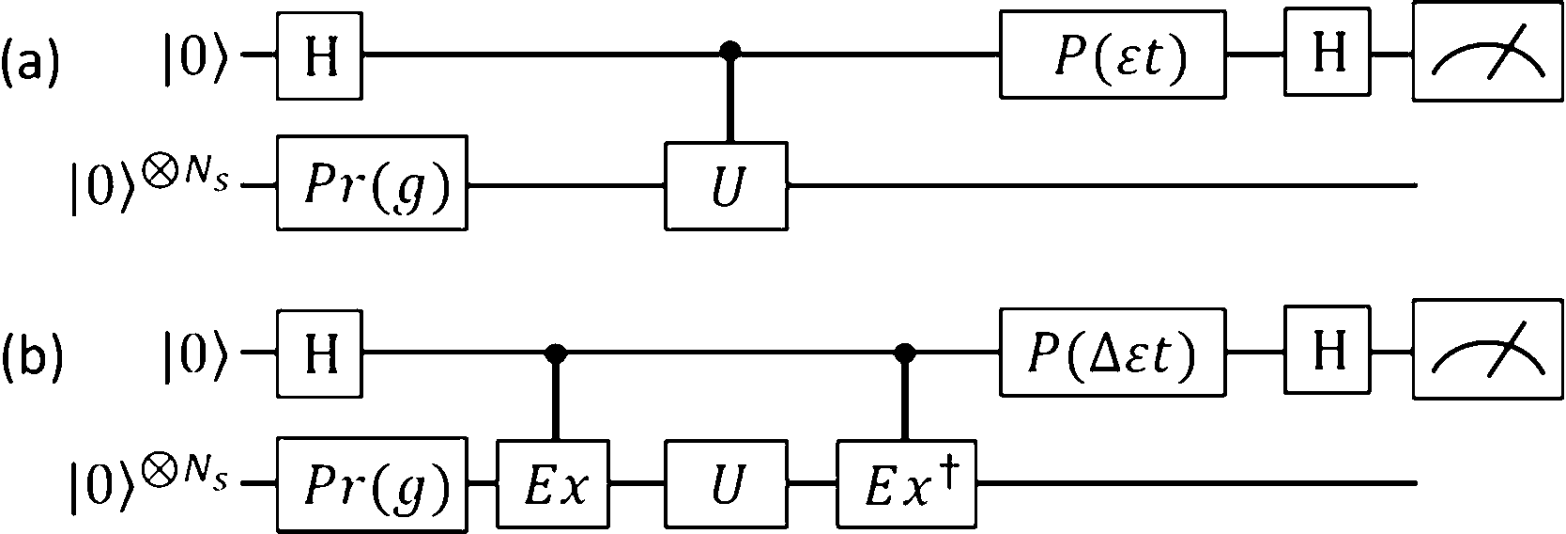
Quantum circuits for (a) BPE and (b) BPDE algorithms. The definition of quantum gates is given in the main text.

First, we briefly discuss the BPE^18–20^ and the BPDE^11^ algorithms. The quantum circuits for the BPE and the BPDE algorithms are shown in Figure 1. Here, H is an Hadamard gate, Pr(*g*) is an approximate ground state preparation gate defined in eq 1, *U* is a time evolution operator given in eq 2, *P* is a phase shift gate defined in eq 3, Ex is a quantum circuit that generates an approximated excited-state wave function |Φ_1_⟩ from the approximated ground-state wave function |Φ_0_⟩ as in eq 4, and ε and Δε are estimators of the total energy and the energy difference, respectively. Throughout this paper, we have used {|Φ⟩} for approximated wave functions and {|Ψ⟩} for eigenfunctions. *N*_s_ in eq 1 is the number of qubits used to store the wave functions. In conventional fermion–qubit mapping methods such as the Jordan–Wigner transformation (JWT)^22^ and the Bravyi–Kitaev transformation (BKT),^23^*N*_s_ is equal to the number of spin orbitals included in the active space. Roughly speaking, QPDE can be implemented by replacing the controlled-time evolution operation in QPE by controlled-excitation and subsequent time evolution and controlled-deexcitation operations.
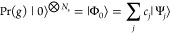
1
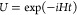
2
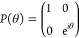
3
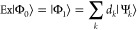
4

By expanding the approximated wave functions by eigenfunctions as given in the right side of eqs 1 and 4, the probability of obtaining the |0⟩ state in the measurement of an ancillary qubit, Prob(0), can be calculated as in eqs 5 and 6 for the BPE and the BPDE, respectively.
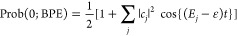
5
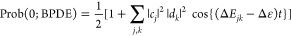
6

These equations insist that if the approximated wave functions |Φ⟩ have sufficiently large overlaps with the eigenfunctions of the target electronic states, then Prob(0) becomes maximum at ε = *E*_*j*_ and Δε = Δ*E*_*jk*_ for the BPE and the BPDE, respectively. In the BPE and the BPDE algorithms, total energies and energy differences are computed by searching for the ε and Δε, respectively, values that give maximum Prob(0), using Bayesian inference.

From eqs 5 and 6, it is clear that the accuracy of the calculated total energies and energy differences in the framework of the BPE and the BPDE algorithms strongly depends on the quality of the approximated wave functions being used as the inputs. If the approximated wave functions are expressed by linear combinations of many eigenstates and contributions from electronic states other than the target state to Prob(0) become non-negligible, then the peak position of Prob(0) may be shifted from that corresponding to the true eigenvalues. For typical closed-shell singlet molecules in their equilibrium geometries, the Hartree–Fock wave function |Φ_HF_⟩ is a reasonably good approximation of the ground-state wave function. However, the overlap squared value |⟨Φ_HF_|Ψ_full–CI_⟩|^2^ can decrease exponentially with the system size, and therefore using |Φ_HF_⟩ as the input wave function is only valid for small molecules. One possible solution is to adopt an adiabatic state preparation (ASP) algorithm to generate correlated wave functions.^2,24–26^ However, the quantum circuit for the ASP is usually deep, and it is unsuitable for the BPE and BPDE frameworks which need repetitive quantum circuit executions.

**Figure 2:**
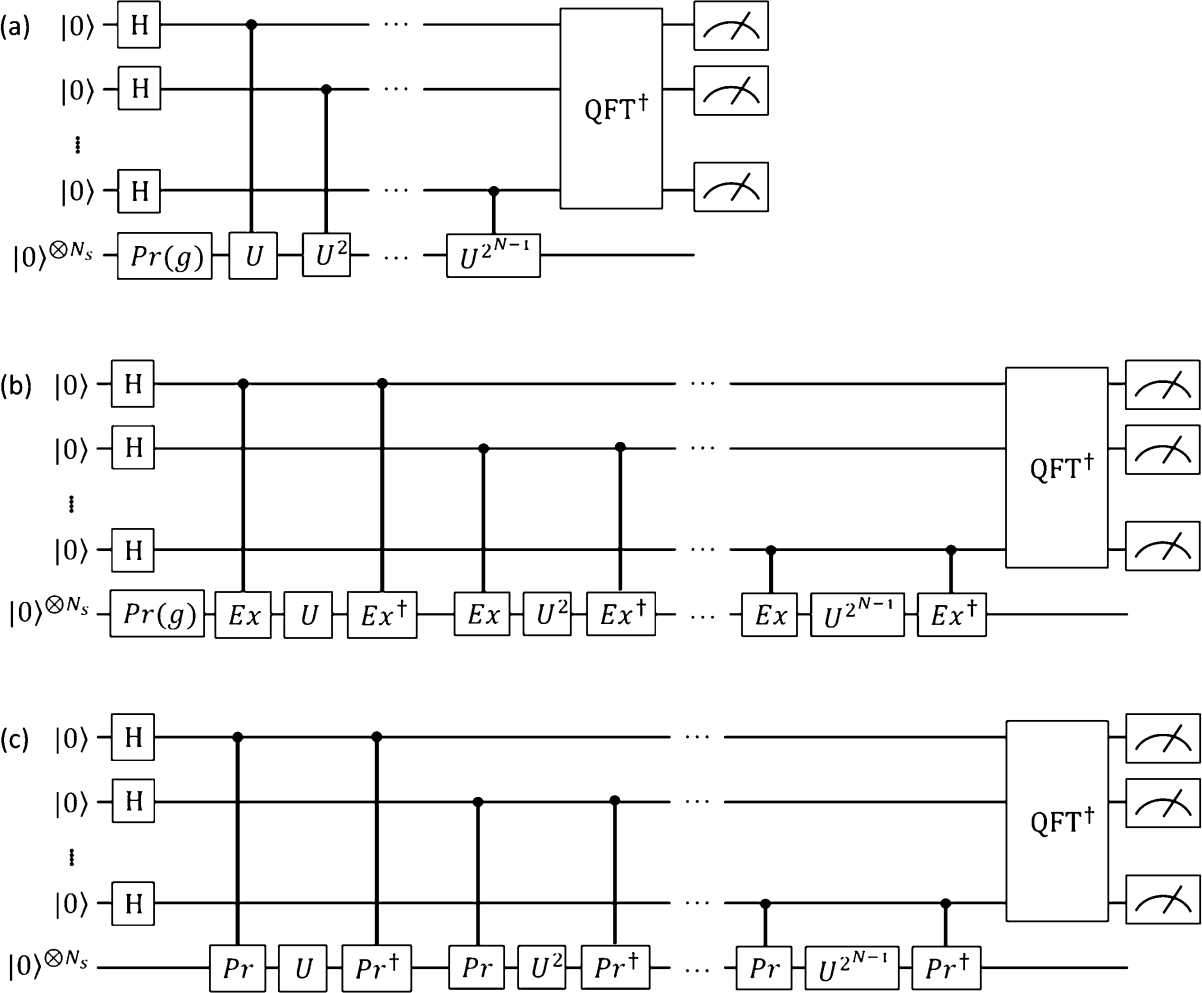
Quantum circuits for (a) *N*-qubit QPE algorithm, (b) *N*-qubit QPDE algorithm in a naive implementation, and (c) *N*-qubit QPDE algorithm used in this work.

In the total energy calculations, single-shot projective calculations are possible by using the QPE algorithm with *N*_a_ of ancillary qubits.^2^ The quantum circuit for the *N*-qubit QPE is shown in Figure 2a. Here, QFT^†^ represents the quantum circuit for the inverse quantum Fourier transformation.^27^ By assuming eq 1, the quantum state before the measurement of ancillary qubits in Figure 2a is calculated as in eq 7.
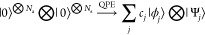
7

Thus, the measurement of ancillary qubits yields an eigenphase ϕ_*j*_ = 0.*x*_1_*x*_2_*x*_3_...*x*_*N*_s__ in binary fraction with the probability proportional to |*c*_*j*_|^2^, and the wave function is projected onto the corresponding eigenfunction |Ψ_*j*_⟩. Here, *x*_*k*_ is the measurement outcome of the *k*-th ancillary qubit. The eigenenergy *E* can be calculated by using the equation *E* = −2πϕ/*t*, which is derived from the equation e^–*iHt*^|Ψ⟩ = e^–*iEt*^|Ψ⟩ = e^2*i*πϕ^|Ψ⟩. From an analogy of the extension of the BPE algorithm to the BPDE, we can construct the quantum circuit for the *N*-qubit QPDE algorithm by replacing the controlled-*U* gates with the sequence of controlled-Ex, *U*, and controlled-Ex^†^ gates, as in Figure 2b. Note that the quantum circuit in Figure 2b contains control-free time evolution operations, and therefore, this implementation implies that |Φ_0_⟩ is an eigenfunction of the time evolution operator. Unfortunately, this assumption is not generally true. This difficulty can be avoided by starting from the |0⟩^⊗*N*_s_^ state and using the controlled-Pr gate given in eq 8 instead of using the controlled-Ex gate, as shown in Figure 2c.
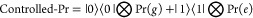
8Here, Pr(*g*)|0⟩^⊗*N*_s_^ = |Φ_0_⟩ and Pr(*e*)|0⟩^⊗*N*_s_^ = |Φ_1_⟩. Since the |0⟩^⊗*N*_s_^ state in JWT and BKT corresponds to the vacuum state with no electrons, applying *U* does not change the state *U*|0⟩^⊗*N*_s_^ = |0⟩^⊗*N*_s_^. As a result, the quantum circuit for the *N*-qubit QPDE is slightly deeper than that for the *N*-qubit QPE. In the present study, the approximated wave functions are expressed by a linear combination of at most two Slater determinants, and thus, the quantum circuits for controlled-Pr are sufficiently shallow. Note that in the present study we have focused on the energy difference of two electronic states at the same molecular geometry, but it is possible to extend the algorithm for the direct calculation of the energy differences of different molecular structures. This can be accomplished by using the time evolution operator conditional on the ancillary qubit (|0⟩⟨0|⊗e^–*iH*_A_*t*^ + |1⟩⟨1|⊗e^–*iH*_B_*t*^) instead of the control-free time evolution operator, where *H*_A_ and *H*_B_ are Hamiltonians at geometries A and B, respectively. If *H*_A_ and *H*_B_ share the same Pauli strings, such a conditional time evolution operation can be implemented by slightly increasing the proportionality factor of the gate count scaling without increasing the scaling of the gate count from the original QPE algorithm by using the technique developed in the BPDE-based numerical energy gradient calculations.^13^

In the implementation described in Figure 2c, the bit string obtained from the measurement of ancillary qubits corresponds to the eigenphase difference Δϕ_*jk*_ = 0.*x*_1_*x*_2_*x*_3_...*x*_*N*_s__, and the eigenenergy difference can be calculated as Δ*E*_*jk*_ = −2πΔϕ_*jk*_/*t*. The probability by which the eigenenergy difference is obtained is proportional to |*c*_*j*_|^2^ × |*d*_*k*_|^2^. Although the overall probability of success in calculating the target energy difference is the same between two separate total energy calculations by the *N*-qubit QPE and the direct calculation of the energy difference by the *N*-qubit QPDE, the identification of the target energy difference may be more difficult for the *N*-qubit QPDE when the quality of the approximated wave functions is not so high. In the case of the total energy calculations, one can determine the ground-state energy based on the variational principle by comparing the obtained energies. In the case of energy difference calculations, however, such an evaluation is not possible. In this context, sophisticated state preparation is more important in the *N*-qubit QPDE algorithm. Nevertheless, the *N*-qubit QPDE-based direct calculation of the energy difference has two advantages over the putative approach based on the *N*-qubit QPE for total energy calculations of individual electronic states: (1) It is free from controlled-time evolution operations, and (2) the number of ancillary qubits can be smaller than in total energy calculations. The latter feature is due to the fact that the energy difference to be discussed is much smaller than the total energy itself. Since the number of ancillary qubits is equal to the number of binary digits of the phase to be determined, molecules with large absolute energies require more qubits.

It should be noted that the energy difference can be positive or negative, but the Δϕ_*jk*_ obtained from the *N*-qubit QPDE quantum circuit does not contain information about the digits above the arithmetic point. Therefore, Δϕ_*jk*_ and Δϕ_*jk*_ – *k*, where *k* is an arbitrary integer, cannot be distinguished in the *N*-qubit QPDE. This means that there is a possibility to assign a wrong sign to the energy difference. To avoid such a misassignment, we can appropriately set the evolution time length *t* so that cos(2πΔϕ_*jk*_) > 0, and use Δϕ_*jk*_ when 0 ≤ Δϕ_*jk*_ ≤ 1/4 and Δϕ_*jk*_ – 1 when 3/4 ≤ Δϕ_*jk*_ < 1. If the eigenphase difference Δϕ_*jk*_ is calculated between 1/4 and 3/4, we cannot immediately determine the sign of the energy difference. However, even in this case, we can determine the sign by performing another *N*-qubit QPDE calculation by adding the phase shift gate *P*(Δ*E*_*jk*_^*l*^) just after time evolution operator *U*^*l*^. This can be understood as follows: When the time evolution operator *U* is applied to the quantum superposition state , the quantum state transforms to , where Δ*E*_01_ = *E*_1_ – *E*_0_. The subsequent application of the phase shift gate results in the quantum state . Thus, if Δ*E*_*jk*_ is positive, the *N*-qubit QPDE with the phase shift gates returns Δϕ = 0, and in the case when Δ*E*_*jk*_ is negative, the *N*-qubit QPDE returns the phase Δϕ = 2Δϕ_*jk*_.

## 3 Computational Conditions

As proof-of-concept demonstrations of the proposed *N*-qubit QPDE algorithm, here we report numerical simulations for the direct calculation of the singlet–triplet energy difference of the H_2_ molecule and the vertical excitation energies of halogen-substituted methylenes (CHF, CHCl, CF_2_, CFCl, and CCl_2_) and formaldehyde (HCHO). We computed the singlet–triplet energy difference as the excitation energy of the spin-triplet state from the spin-singlet state. Because all of the molecules under study have the spin-singlet ground state, the singlet–triplet energy difference Δ*E*_ST_ = *E*(T_1_) – *E*(S_0_) is positive. Note that numerical simulations of the BxB and the BPDE-based energy difference calculations have been reported^10,11^ for some of the compounds under study. In our previous publications, the singlet–triplet energy difference of H_2_ was defined as Δ*E* = *E*(S_0_) – *E*(T_1_), and therefore, the definition of the energy difference is different. The one- and two-electron integrals required to construct the electronic Hamiltonian were computed by using the *General Atomic and Molecular Electronic Structure System* (GAMESS-US) program package.^28^ The full-CI and the complete active space configuration interaction (CAS-CI) calculations as the references were also performed with GAMESS-US. Numerical quantum circuit simulations were performed by using our in-house Python code developed with the OpenFermion^29^ and Cirq^30^ libraries.

The singlet–triplet energy difference of the H_2_ molecule with the atom–atom distance from 0.7 to 3.0 Å was studied by using the 6-31G basis set. The wave function is encoded in 8 qubits using JWT, and 12 ancillary qubits were used for the phase readout. The evolution time length in the *U* = exp(−*iHt*) operator was set to *t* = 10. We set the evolution time length *t* considerably long because the singlet–triplet energy difference of the H_2_ molecule is very small for long H–H distances. The second-order Trotter–Suzuki decomposition^31,32^ given in eq 9 was adopted to construct the quantum circuit. An error of the second-order Trotter decomposition scales quadratic against the time length of the single Trotter step Δ*t*,^33,34^ which is very important to apply the AEM. We examined three different time lengths for the single Trotter steps: Δ*t* = *t*/M = 0.5, 1.0, and 1.25.
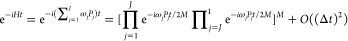
9Here, *P*_*j*_ is a Pauli string described by a direct product of Pauli operators {*I*, *X*, *Y*, *Z*}, ω_*j*_ is the corresponding coefficient, and *J* is the number of Pauli strings included in Hamiltonian *H*. It is known that the Trotter–Suzuki decomposition error depends on the term ordering.^35,36^ In this study, we used a magnitude ordering^35^ where the Pauli string is applied in decreasing order of |ω_*j*_|.

**Figure 3:**
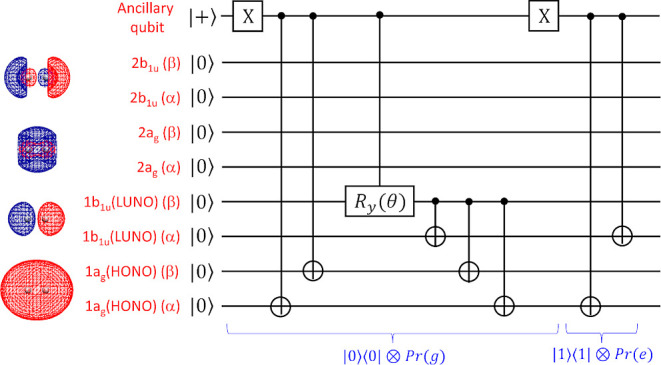
Natural orbitals constructed from the BS-UHF wave function and the controlled-Pr gate used for the *N*-qubit QPDE calculations of the H_2_ molecule. The rotation angle of the *R*_*y*_ gate is set to be , where *y* is the diradical character calculated using eq 11.

As discussed in the previous section, the probability of which energy difference can be obtained is proportional to |*c*_*j*_|^2^ × |*d*_*k*_|^2^, where *c*_*j*_ and *d*_*k*_ are the coefficients defined in eqs 1 and 4, respectively. This means that the accuracy of the approximated wave functions only affects the success probability, and if success occurs, the energy difference obtained from the *N*-qubit QPDE does not depend on the quality of the wave functions. To study the input wave function dependence in a H_2_ molecule with the 6-31G basis set, we used two different wave functions for the ground-state spin-singlet wave functions. One is the Hartree–Fock wave function |Φ_HF_⟩, and the other is the two-configurational wave function |Φ_2*c*_⟩ constructed by using a diradical character *y* and the natural orbitals computed at the broken-symmetry spin-unrestricted Hartree–Fock (BS-UHF) level. As reported in our precedent paper,^37^ the |Φ_2*c*_⟩ defined as in eq 10 can be a qualitatively good approximated wave function of diradical systems with a large overlap with the full-CI wave function. The diradical character *y* was calculated from the occupation number of the lowest unoccupied natural orbital (*n*_LUNO_) constructed from the BS-UHF wave function for the *M*_S_ = 0 state, as given in eq 11.^38^
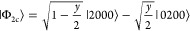
10
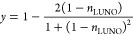
11Here, |2000⟩ is the Hartree–Fock (HF)-like configuration  and |0200⟩ is the highest occupied natural orbital (HONO)–lowest unoccupied natural orbital (LUNO) two-electron excited determinant from the HF-like configuration in the H_2_ molecule with the 6-31G basis set (4 molecular orbitals and 2 electrons). The BS-UHF natural orbitals of H_2_ are illustrated in Figure 3. For the spin-triplet state, we used the spin-restricted open-shell Hartree–Fock (ROHF)-like configuration |Φ_1_⟩ = |αα00⟩, where α indicates that the molecular orbital is singly occupied by a spin-α electron. The controlled-Pr gates used for the calculations are depicted in Figure 3.

The numerical simulations of halogen-substituted methylenes were carried out at the CAS-CI/6-31G* level using the Becke, 3-parameter, Lee–Yang–Parr (B3LYP)/6-31G* optimized geometry. Geometry optimizations were performed using Gaussian 09 software.^39^ Cartesian coordinates of the optimized geometries are summarized in the Supporting Information. We used the (6e, 4o) active space for CHF and CHCl and the (10e, 6o) active space for CF_2_, CFCl, and CCl_2_, and the vertical excitation energies of the lowest spin-triplet excited state (1^3^B_1_ state) were calculated. Here, (*k*e, *l*o) specifies that the active space contains *k*-electrons and *l*-molecular orbitals. All the active spaces are given as Figures S1–S5 in the Supporting Information. We used ten ancillary qubits for the phase readout, and the evolution time length was set to be *t* = 10. The restricted Hartree–Fock (RHF) and ROHF-like single determinant wave functions were used for |Φ_0_⟩ and |Φ_1_⟩, respectively, and the quantum circuit similar to Figure 3 with θ = 0 is used for the controlled-Pr gate.

For direct calculations of the vertical excitation energies of formaldehyde (HCHO), we focused on the three low-lying excited states (1^1^A_2_, 1^1^B_1_, and 2^1^A_1_ states). We used the same geometry and active space as our previous publication.^11^ The CAS-CI active space consists of 5a_1_ (C–O σ), 1b_1_ (C–O π), 2b_2_ (in-plane 2p lone pair of O), 2b_1_ (C–O π*), and 9a_1_ (C–O σ*) orbitals, as illustrated in Figure S6 in the Supporting Information. The excited-state wave functions are approximated by the spin symmetry-adapted (2b_2_ → 2b_1_), (5a_1_ → 2b_1_), and (1b_1_ → 2b_1_) one-electron excitations from the |Φ_HF_⟩ for the 1^1^A_2_, 1^1^B_1_, and 2^1^A_1_ states, respectively. The quantum circuits of the controlled-Pr gates are illustrated in Figure S7 in the Supporting Information. The simulations were carried out using ten ancillary qubits and *t* = 10.

## 4 Results and Discussion

### 4.1 Effect of Input Wave Function in H_2_ Molecule

First, we checked the effect of the approximated wave functions on the success probability of the *N*-qubit QPDE and the calculated eigenphase difference value, by using |Φ_HF_⟩ and |Φ_2*c*_⟩ as the input wave functions for the spin-singlet electronic ground state of the H_2_ molecule. We used two different geometries *R*(H–H) = 2.0 and 3.0 Å as the representative examples of intermediate bond-breaking and bond-dissociation regions, respectively. The ground-state wave function exhibits a sizable diradical character [*y* = 0.4398 and 0.8648 for *R*(H–H) = 2.0 and 3.0 Å, respectively], and therefore |Φ_2*c*_⟩ is a much better approximation for the ground-state wave function than |Φ_HF_⟩.

**Figure 4:**
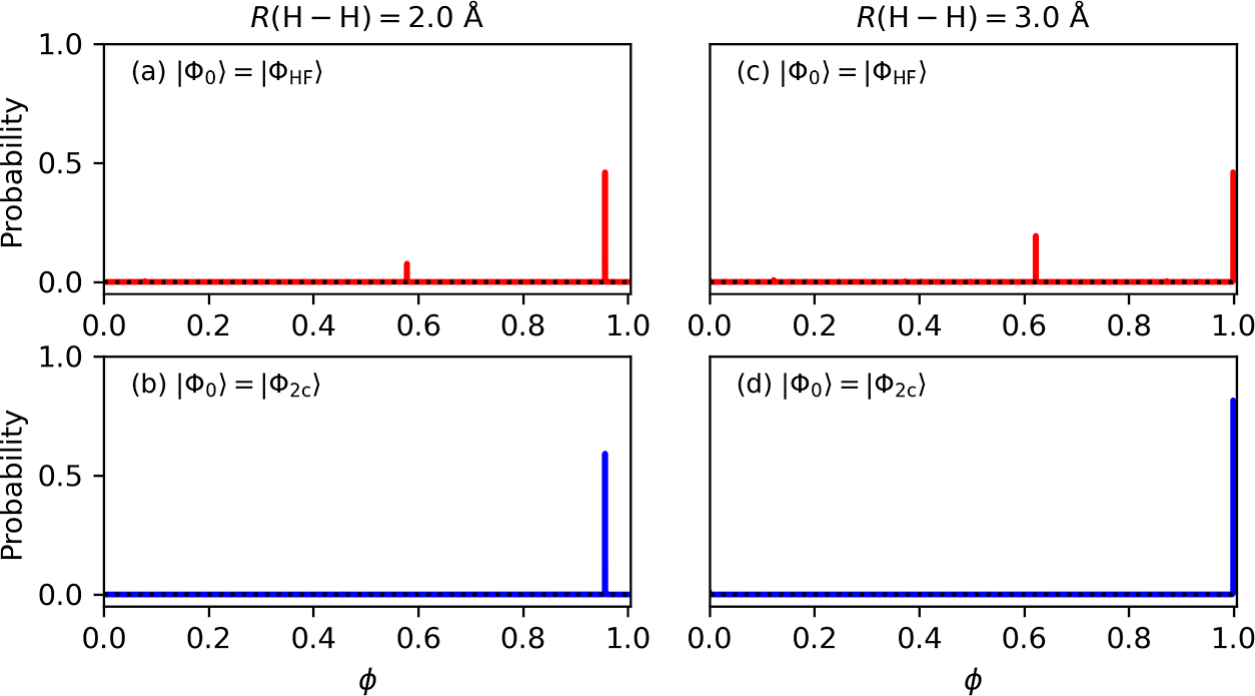
Phase value vs probability plot obtained from the *N*-qubit QPDE simulations of H_2_ molecules with different input wave functions for the S_0_ ground state.

Figure 4 summarizes the relationship between the phase value obtained from the measurement of the ancillary qubits and the probability of occurrence. The plots have two major peaks when |Φ_HF_⟩ is used as the input wave function for the singlet ground state. By converting the phase values of the peaks at the geometry *R*(H–H) = 2.0 Å to the energy unit, we obtained the energy differences as 0.027305 and −0.363553 hartree for the major and minor peaks, respectively. These values correspond to the (T_1_ – S_0_) and (T_1_ – S_2_) energy differences [Δ*E*(full-CI/6-31G) = 0.027980 and −0.361740 hartree, respectively]. Importantly, the difference in the input wave functions only affects the success probability, and the phase value giving a peak remains unchanged. These results support the projective nature of the *N*-qubit QPDE algorithm.

To check the consistency of the calculated energy differences when summed, we performed additional simulations for the direct calculation of the (S_2_–S_0_) excitation energy. The (S_2_–S_0_) energy difference calculated directly from the *N*-qubit QPDE is 0.390858 hartree, which is identical to the (T_1_–S_0_) – (T_1_–S_2_) value.

### 4.2 Singlet–Triplet Energy Difference of H_2_ Molecule with Different Bond Lengths and Application of the Algorithmic Error Mitigation

**Figure 5:**
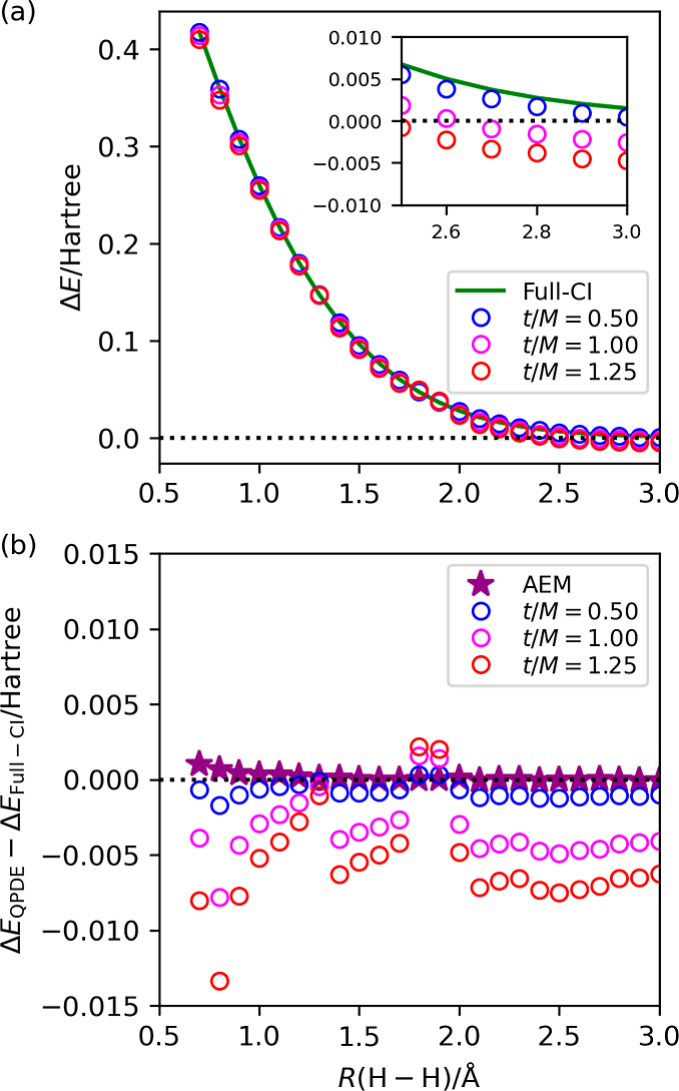
*N*-qubit QPDE simulation results of the H_2_ molecule with different time lengths of the single Trotter step *t*/*M*. (a) Singlet–triplet energy difference. Inset is the plot of Δ*E* at the geometry *R*(H–H) ≥ 2.5 Å. (b) Differences between the singlet–triplet energy differences from the quantum circuit simulations and from the full-CI/6-31G calculations. AEM stands for the energy difference calculated by using the algorithmic error mitigation technique (see text).

Next, we carried out the *N*-qubit QPDE simulations for the direct calculation of the singlet–triplet energy difference of the H_2_ molecule by changing the atom–atom distance from 0.7 to 3.0 Å. The calculated energy differences are plotted in Figure 5a and the deviations from the full-CI/6-31G energy difference are given in Figure 5b. Here, we used |Φ_2*c*_⟩ as the input wave function of the spin-singlet state, and the phase value giving the maximum measurement probability was taken as the eigenphase. From Figure 5, it is clear that the deviation from the full-CI value becomes smaller when a shorter time length is adopted for the single Trotter step. For the geometry with longer *R*(H–H) distances, the *N*-qubit QPDE simulations with *t*/*M* = 1.00 and 1.25 gave negative Δ*E* values (see the inset of Figure 5a), which are in contradiction with the full-CI/6-31G results.

The plot in Figure 5b indicates that the Δ*E* values obtained from the *N*-qubit QPDE simulations have systematic errors. We assume that the main source of the errors is the Trotter–Suzuki decomposition. Apart from the Trotter–Suzuki decomposition error, a rounding error of the phase value due to the finite number of ancillary qubits can affect the Δ*E* values. Theoretically, using a shorter time length for the single Trotter step can systematically reduce the error, but there is a trade-off between the Trotter–Suzuki decomposition error and the computational cost. Instead of using a shorter time length for the single Trotter step, here we examined the AEM method.^21^ The AEM is a technique for mitigating errors of algorithmic origin such as the Trotter–Suzuki decomposition. It is known that the error in the second-order Trotter–Suzuki decomposition scales as *O*((Δ*t*)^2^),^33,34^ as given in eq 9. Since we have fixed the evolution time length *t* for different Δ*t* = *t*/*M* simulations, the energy difference including the Trotter–Suzuki decomposition error can be fitted by a quadratic function, *f*(Δ*t*) = *a*(Δ*t*)^2^ + *b*, where *f*(Δ*t*) is the energy difference obtained from the *N*-qubit QPDE simulation with the single Trotter step size *t*/*M* = Δ*t*. The residue *b* of the fitted function corresponds to the energy difference of the Trotter–Suzuki decomposition error zero limit estimated from the extrapolation. The results of the AEM are also plotted in Figure 5b (purple stars). It is clear that the AEM efficiently reduces the error in the energy difference estimation. In the case of the H_2_ molecule, the maximum value of |Δ*E* (AEM) – Δ*E* (full – CI)| is 0.00101 hartree = 0.633 kcal mol^–1^, which is below the chemical precision (1.0 kcal mol^–1^).

### 4.3 Vertical Lowest-Triplet Excitation Energies of Halogen-Substituted Methylenes

Next, we focused on the vertical excitation energy of the lowest spin-triplet state (1^3^B_1_ state) of halogen-substituted methylenes (CHF, CHCl, CF_2_, CFCl, and CCl_2_). Note that in the precedent paper we reported the direct calculation of the vertical excitation energies of CF_2_ and CCl_2_ molecules using the BPDE algorithm.^11^

**Figure 6:**
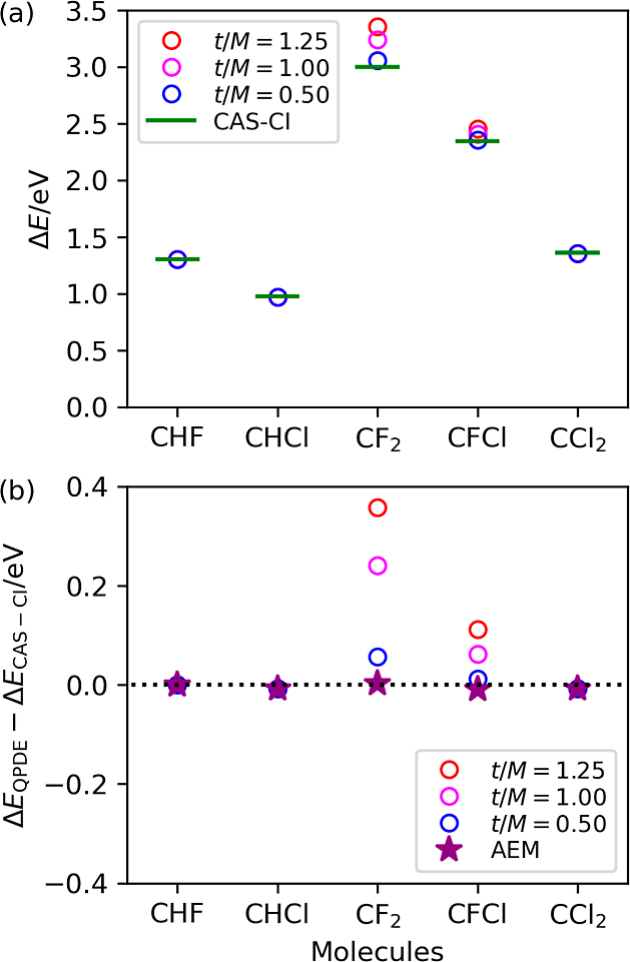
*N*-qubit QPDE simulation results of the vertical lowest-triplet excitation energies of halogen-substituted methylenes with different time lengths of the single Trotter step *t*/*M*. (a) Excitation energy. (b) Differences between the excitation energies from the quantum circuit simulations and those from the CAS-CI/6-31G* calculations. AEM stands for the energy difference obtained by applying algorithmic error mitigation.

The results of the *N*-qubit QPDE simulations are plotted in Figure 6. We observed a strong system dependence of the Trotter–Suzuki decomposition errors. In CHF, CHCl, and CCl_2_ molecules, the *N*-qubit QPDE simulations with three different *t*/*M* values yielded the same energy difference, and the calculated energy differences agree with the CAS-CI values with less than 0.01 eV of deviation. In contrast, the Trotter–Suzuki decomposition errors are significant for CF_2_ and CFCl molecules. For example, the deviations of the excitation energies obtained from the *N*-qubit QPDE from the CAS-CI values in CF_2_ are 0.056, 0.240, and 0.357 eV for *t*/*M* = 0.50, 1.00, and 1.25, respectively. However, by adopting the AEM to mitigate the Trotter–Suzuki decomposition error, the departure of the excitation energy from the CAS-CI value becomes 0.002 eV. The deviations of the Δ*E*_QPDE_ (AEM) – Δ*E*_CAS–CI_ of CHF, CHCl, CFCl, and CCl_2_ were calculated to be 0.0000, −0.0075, −0.0096, and −0.0075 eV, respectively. These results also illustrate the importance of the AEM in accurately predicting excitation energies using the *N*-qubit QPDE algorithm.

To check the dependence of the evolution time length *t* on the accuracy of the calculated vertical excitation energies, we also carried out *N*-qubit QPDE simulations with *t* = 5 instead of *t* = 10. The results are shown in Figure S8 in the Supporting Information. The calculated Δ*E*_QPDE_ (AEM) – Δ*E*_CAS–CI_ values are 0.0000, −0.0075, 0.0259, 0.0141, and 0.0117 eV for CHF, CHCl, CF_2_, CFCl, and CCl_2_, respectively. Interestingly, when the Trotter–Suzuki decomposition error is small, as in CHF and CHCl, using a shorter evolution time does not affect the calculated excitation energies. However, when the Trotter–Suzuki decomposition error becomes significant, as in CF_2_, CFCl, and CCl_2_, the error in the Δ*E*_QPDE_ (AEM) also becomes large. We expect that the rounding error on the phase value for each simulation will increase with a shorter evolution time, which will affect the performance of the AEM.

### 4.4 Vertical Excitation Energies of Formaldehyde

**Figure 7:**
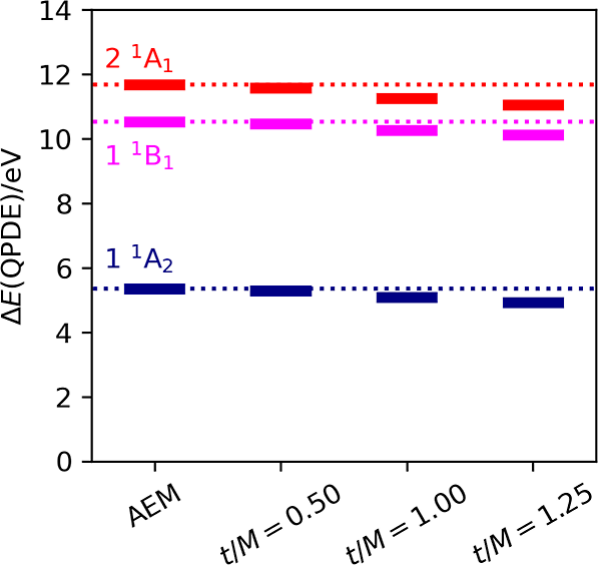
*N*-qubit QPDE simulation results of the vertical excitation energies of formaldehyde with different time lengths of the single Trotter step *t*/*M*. AEM stands for the excitation energies obtained by applying the algorithmic error mitigation, and dotted horizontal lines indicate the CAS-CI excitation energies.

The two examples above deal with the energy difference between the lowest singlet and the lowest triplet states. Here, we focus on the vertical excitation energies of the spin-singlet excited states of formaldehyde (HCHO). We have calculated the excitation energies of three low-lying states: the 1^1^A_2_, the 1^1^B_1_, and the 2^1^A_1_ states. As discussed in the Computational Conditions section, these excited states can be approximated by the spin symmetry-adapted (2b_2_ → 2b_1_), (5a_1_ → 2b_1_), and (1b_1_ → 2b_1_) one-electron excitations, respectively, from the |Φ_HF_⟩ state. Note that direct calculations of the excitation energies of these states have been reported by us using the BPDE algorithm.^11^ The results are summarized in Figure 7. Again, the Trotter–Suzuki decomposition causes systematic error in the excitation energies, but the error can be efficiently removed by applying the AEM. The calculated excitation energies and those obtained from the BPDE simulations^11^ are listed in Table 1. Importantly, in all of the excited states under study, the excitation energies obtained from the *N*-qubit QPDE in conjunction with the AEM agreed with the CAS-CI values with less than 0.006 eV = 0.138 kcal mol^–1^ of deviation, which is smaller than that of the Δ*E*_BPDE_ values listed in Table 1. Note that the BPDE simulations^11^ were carried out using *t*/*M* = 0.50. The excitation energies calculated using the *N*-qubit QPDE and the BPDE methods with the same *t*/*M* = 0.50 value are very close to each other. The differences in the excitation energies between the two calculations are mainly due to the shot noise in the BPDE simulations and the nonprojective nature of the BPDE algorithm.

**Table 1:** Vertical Excitation Energies of Formaldehyde Calculated by Using the *N*-qubit QPDE and the BPDE Algorithms and at the CAS-CI Level of Theory

state	Δ*E*_QPDE_/eV				Δ*E*_BPDE_/eV^b^	Δ*E*_CAS__–__CI_/eV
	*t*/*M* = 1.25	*t*/*M* = 1.00	*t*/*M* = 0.50	AEM^a^		
1^1^A_2_	4.942	5.092	5.293	5.360	5.297	5.359
1^1^B_1_	10.135	10.268	10.469	10.530	10.466	10.525
2^1^A_1_	11.053	11.270	11.588	11.686	11.603	11.692

a The energy difference obtained by applying the algorithmic error mitigation.

b Ref (11).

## 5 Conclusions

In this work, we developed a quantum phase difference estimation algorithm with *N*_a_ of ancillary qubits for the direct calculations of energy differences on a quantum computer. Although the number of ancillary qubits required to run the algorithm is larger than the previously proposed Bayesian inference-based implementations,^11–14^ the proposed approach is based on the projective measurement, and therefore it is able to calculate the difference of energy eigenvalues of two electronic states with the approximated wave functions as inputs. The quality of the approximated wave functions only affects the success probability of the calculation, and the calculated energy difference value is independent of the input wave functions. The calculated energy differences show systematic errors mainly caused by the Trotter–Suzuki decomposition. We demonstrated that the error is effectively reduced by applying the algorithmic error mitigation technique. For all the molecules being studied, the error-mitigated energy difference agreed to the full-CI or CAS-CI reference value with less than 1 kcal mol^–1^ of deviations. Since sophisticated wave function preparation methods such as an adiabatic state preparation require a deep quantum circuit, the single-shot protocol developed in this work is suitable to connect with such approaches. The *N*-qubit QPDE method with the input wave functions generated by using ASP is in progress and will be discussed in the forthcoming paper.

## Supporting Information Available

Cartesian coordinates and CAS-CI active spaces of halogen-substituted methylenes and formaldehyde, controlled-Pr gates used for the excitation energy calculations of formaldehyde, and *N*-qubit QPDE simulation results of the vertical excitation energies of halogen-substituted methylenes with the evolution time length *t* = 5 (PDF)
