# Supplementary material for: Projective Measurement-Based Quantum Phase Difference Estimation Algorithm for the Direct Computation of Eigenenergy Differences on a Quantum Computer
Source: J Chem Theory Comput. 2023 Oct 24;19(21):7617–25. doi: 10.1021/acs.jctc.3c00784 (PMC10653105; doi:10.1021/acs.jctc.3c00784)
Supplement: Supplementary file 1 — ct3c00784_si_001.pdf [file ct3c00784_si_001.pdf]

Supporting Information

Projective Measurement-Based Quantum Phase Difference Estimation Algorithm for the Direct Computation of Eigenenergy Differences on a Quantum Computer

Kenji Sugisaki[1,2,3]*

[1] *Graduate School of Science and Technology, Keio University, 7-1 Shinkawasaki, Saiwai-ku, Kawasaki, Kanagawa 212-0032, Japan.*

[2] *Quantum Computing Center, Keio University, 3-14-1 Hiyoshi, Kohoku-ku, Yokohama, Kanagawa 223-8522, Japan*

[3] *Centre for Quantum Engineering, Research and Education (CQuERE), TCG Centres for Research and Education in Science and Technology (TCG CREST), Sector V, Salt Lake, Kolkata 700091, India*

* E-mail: ksugisaki@keio.jp

Cartesian coordinates of the UB3LYP/6-31G* optimized geometry of CHF in units of Å.

H	−1.039570	0.955273	0.000000
C	0.069305	0.726085	0.000000
F	0.069305	−0.590198	0.000000

Cartesian coordinates of the UB3LYP/6-31G* optimized geometry of CHCl units of Å.

H	−1.050282	1.436131	0.000000
C	0.045664	1.214993	0.000000
Cl	0.045664	−0.513299	0.000000

Cartesian coordinates of the UB3LYP/6-31G* optimized geometry of CF_2 in units of Å.

F	0.000000	1.035667	−0.201987
C	0.000000	0.000000	0.605962
F	0.000000	−1.035667	−0.201987

Cartesian coordinates of the UB3LYP/6-31G* optimized geometry of CFCl in units of Å.

F	1.291310	0.770137	0.000000
C	0.000000	0.922169	0.000000
Cl	−0.683635	−0.733191	0.000000

Cartesian coordinates of the UB3LYP/6-31G* optimized geometry of CCl_2 in units of Å.

Cl	0.000000	1.429977	−0.152156
C	0.000000	0.000000	0.862218
Cl	0.000000	−1.429977	−0.152156

Cartesian coordinates of the UB3LYP/6-31G* optimized geometry of HCHO in units of Å.

O	0.000000	0.000000	0.677534
C	0.000000	0.000000	−0.528862
H	0.000000	0.937777	−1.123552
H	0.000000	−0.937777	−1.123552

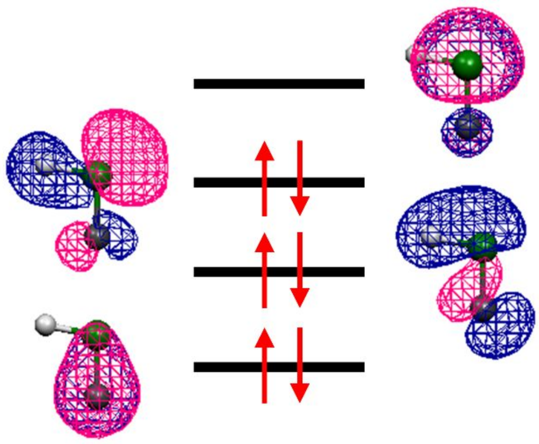

Figure S1: Active space of CHF.

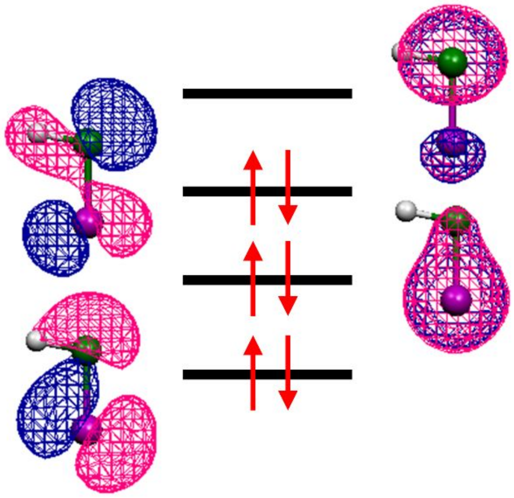

Figure S2: Active space of CHCl

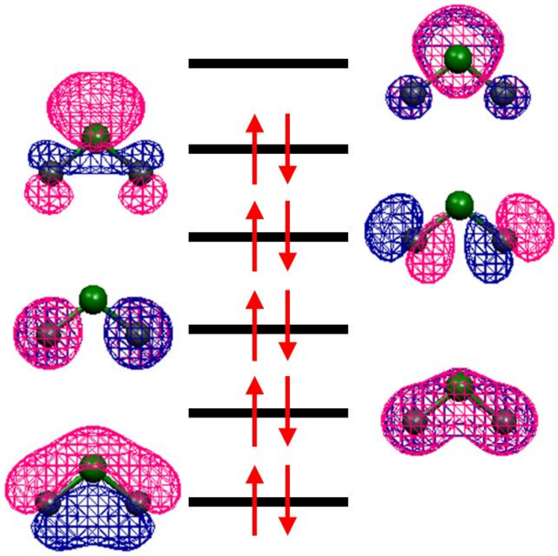

Figure S3: Active space of CF_2

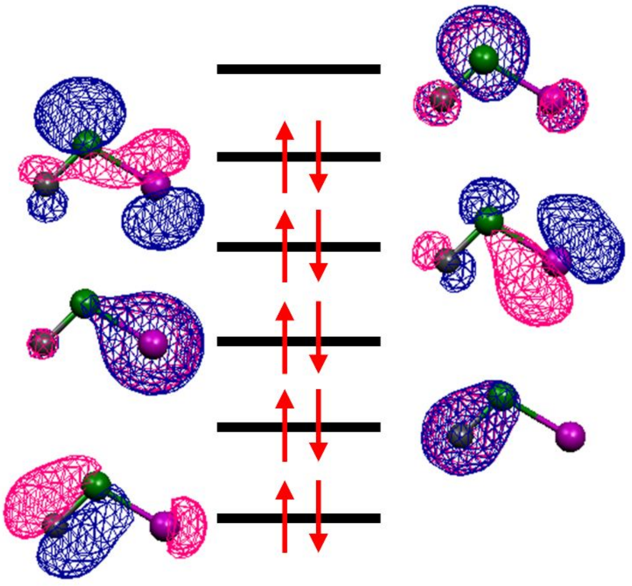

Figure S4: Active space of CFCl

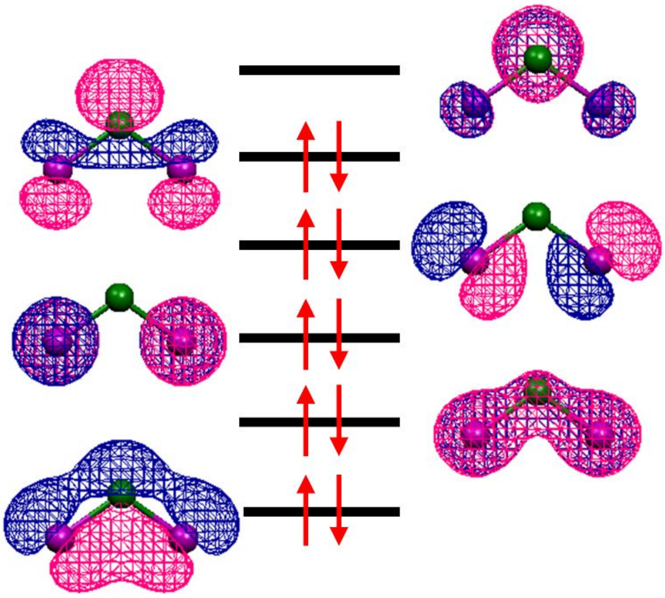

Figure S5: Active space of CCl_2

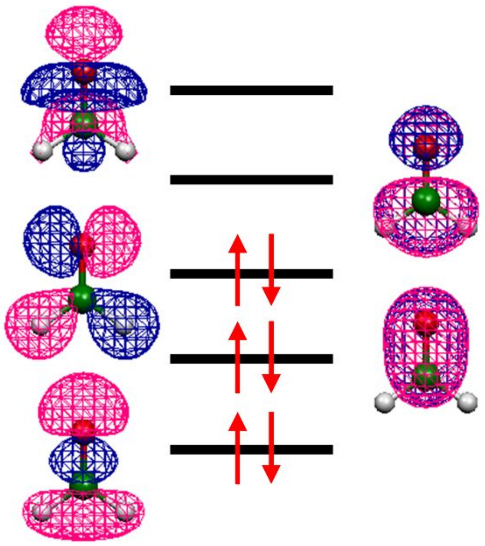

Figure S6: Active space of HCHO.

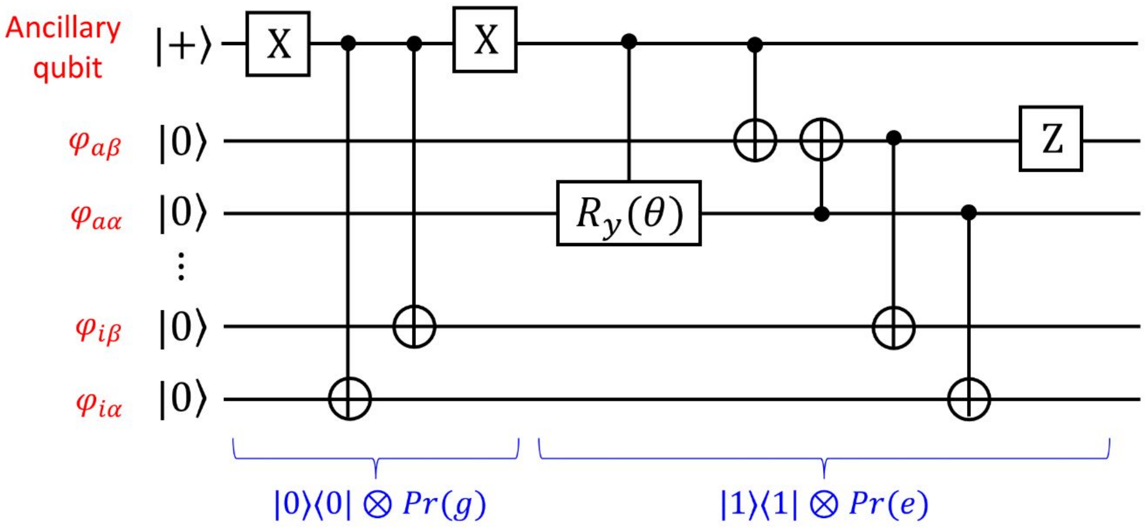

Figure S7: The controlled-*Pr* gate used for the *N*-qubit QPDE simulations of formaldehyde. The excited state wave function is approximated by the spin symmetry adapted one electron excitation from *i*-th occupied orbital to *a*-th virtual orbital. Qubits corresponding to spin orbitals other than *i*-th occupied and *a*-th virtual orbitals are omitted for clarity. The Pauli-X gate should be applied to the qubits corresponding to the doubly occupied orbitals those do not appear in the Figure.

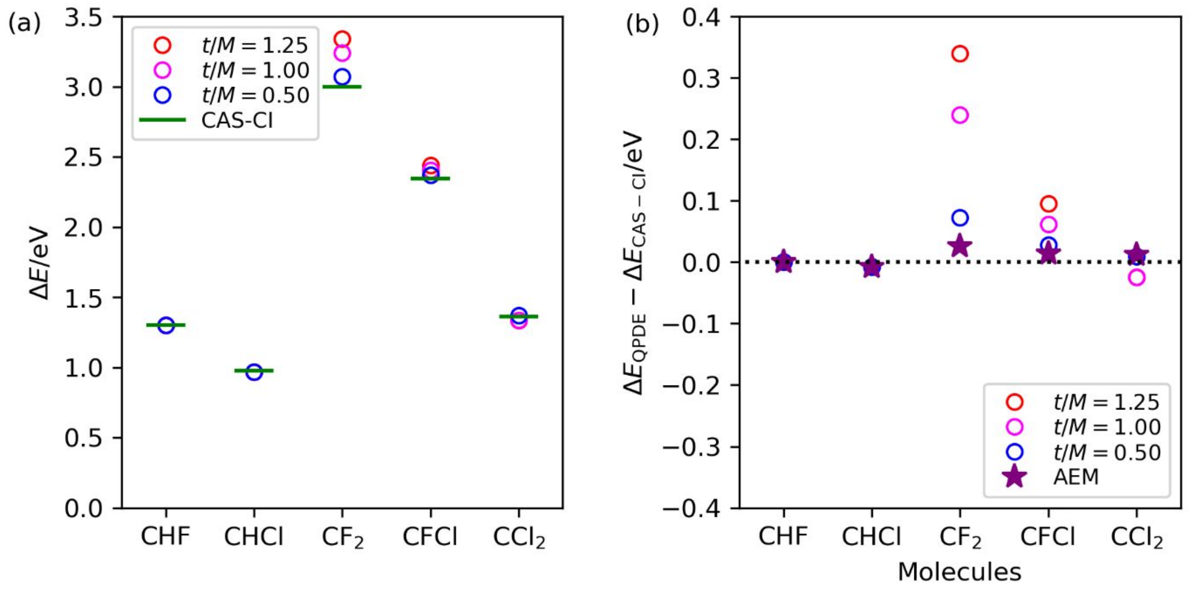

Figure S8: The *N*-qubit QPDE simulation results of the vertical lowest triplet excitation energies of halogen-substituted methylenes with different time lengths of the single Trotter step t/M and the evolution time length t = 5. (a) The excitation energy. (b) Differences between the excitation energies from the quantum circuit simulations and those from the CAS-CI/6-31G* calculations. AEM stands for the excitation energy obtained by applying the algorithmic error mitigation.
